# Evolutionary de Winter pattern: from de Winter ECG to STEMI-A case report

**DOI:** 10.1186/s12872-020-01611-0

**Published:** 2020-07-06

**Authors:** Hao Wang, Xiao-Ce Dai, Yun-Tao Zhao, Xiao-Hang Cheng

**Affiliations:** 1 Department of Coronary Care Unit, Beijing Royal Integrative Medicine Hospital, No. 1, Wangfu street, Changping District, Beijing, 102200 P.R. China; 2 Department of Cardiology, Affiliated Hospital of Jiaxing University, No.1882 Zhonghuan South Road, Jiaxing, Zhejiang, 314000 P.R. China; 3 Department of Cardiology, Aerospace Center hospital, 15 Yuquan road, Beijing, 100049 P.R. China

**Keywords:** Cardiac anatomy, Noninvasive, Case report

## Abstract

**Background:**

De Winter pattern is associated with acute occlusion in the left anterior descending coronary artery combined with upsloping ST-segment depression at the J point in leads V_1_ through V_6_ without ST-segment elevation. The ECG changes in this case were illustrated by an up-sloping ST-segment depression in the V_1_ to V_6_ leads, followed by tall and symmetrical T waves. Changes from de Winter to ST-segment elevation myocardial infarction (STEMI) are rare.

**Case presentation:**

Our case illustrated an evolutionary de Winter sign that changed to STEMI; the patient underwent cardiac catheterization in time.

**Conclusions:**

Patients who have an electrocardiogram showing de Winter changes may require primary percutaneous coronary intervention. Emergency physicians and cardiologists should not ignore these changes.

## Background

Acute subtotal or total proximal left anterior descending (LAD) occlusion can present de Winter ST-T changes. The main characteristics of the de Winter electrocardiogram (ECG) pattern are up-sloping ST-segment depression in the V_1_ to V_6_ leads, followed by tall and symmetrical T waves [1], which remain consistent with no evolutionary ECG changes. We present a patient with acute proximal LAD occlusion who presented with an evolutionary ECG change in which the de Winter ST-T changes evolved into ST-segment elevation myocardial infarction (STEMI).

## Case presentation

**Fig. 1:**
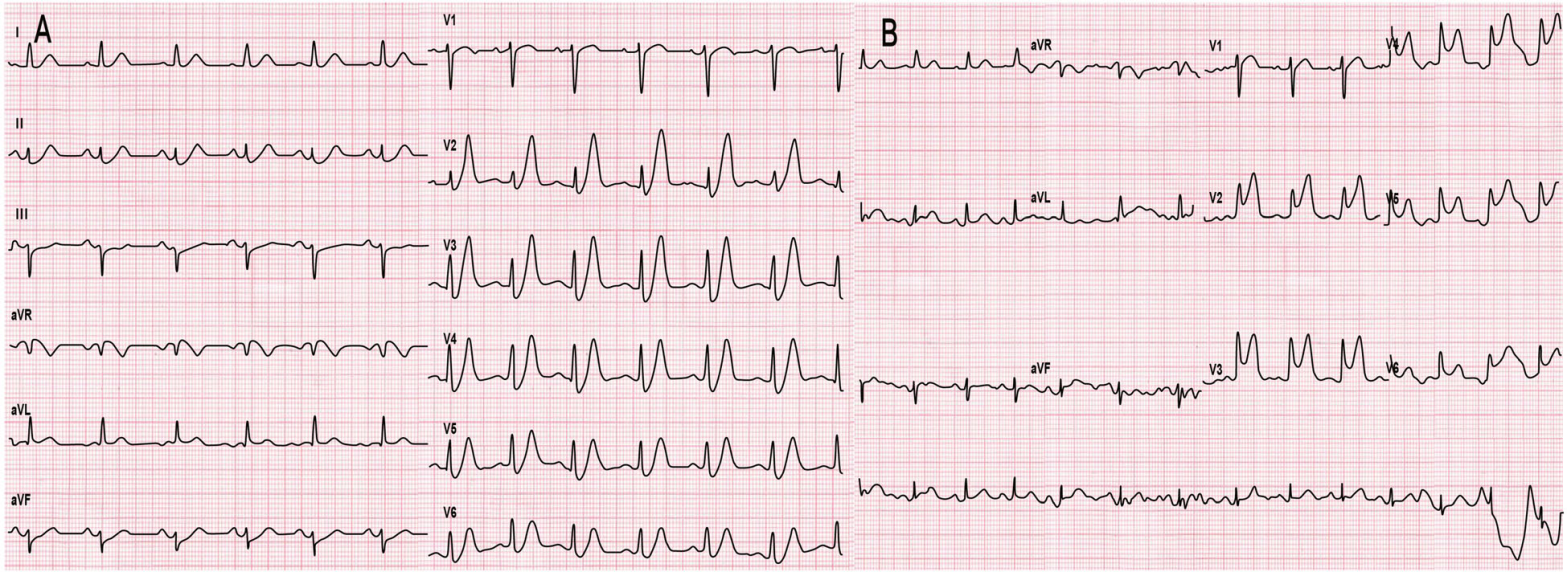
**a** The first ECG revealed up-sloping ST-segment depression in the V_2_ to V_6_ leads, followed by tall and symmetrical T waves in the precordial leads; **b** the second ECG performed 30 min after admission showed ST-segment elevation in lead V_2_ toV_6_

**Fig. 2:**
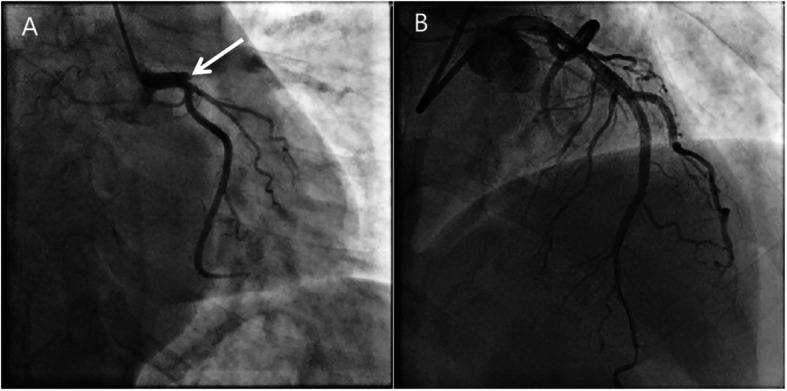
**a** Coronary angiogram revealed a total occlusion in the proximal LAD ([white arrowhead]; **b** The lesion was treated by PCI with one drug-eluting stent, showing TIMI-3 flow

A 31-year-old male with no medical history presented to the emergency department with persistent chest pain for 1 h. The first ECG (finished in the ambulance) revealed up-sloping ST-segment depression in the V_2_ to V_6_ leads, followed by tall and symmetrical T waves in the precordial leads, characterized as de Winter ST-T changes (Fig. 1a). The initial troponin I level was 1.48 μg/L (reference range, 0–0.4 μg/L). The patient received dual loading antiplatelet and statin therapy (aspirin 300 mg, ticagrelor 180 mg and rosuvastatin 20 mg). Thirty minutes later, the arrival ECG showed ST-segment elevation in lead V_2–6_ (Fig. 1b), and the troponin I level increased to 3.68 μg/L. The patient underwent primary percutaneous coronary intervention (PCI), revealing a total occlusion in the proximal LAD (TIMI-0 flow; Fig. 2a) and no stenosis was found in the right or left circumflex coronary arteries. Stent implantation of the LAD artery was performed successfully (Fig. 2b).

## Discussion

Our case illustrated an atypical example of the de Winter ECG pattern subsequently developing into the STEMI pattern due to coronary occlusion. We considered this phenomenon to be related to thrombotic instability in the proximal segment of the LAD. Therefore, we propose that the de Winter ECG pattern should be classified into two categories: (1) the static type: a J-point depression persisting until the reperfusion of the LAD with no evolution into STEMI; and (2) the evolutionary ECG change type: a mutual conversion between STEMI and de Winter ST-T changes, which depends on the state of the LAD between complete occlusion and spontaneous recanalization [2, 3].

Approximately 2% of patients with acute subtotal or total occlusion of the proximal LAD artery do not have the usual ST-segment elevation but instead have the de Winter ECG pattern, which was first described as acute subtotal or total proximal LAD occlusion in 2008 in the New England Journal of Medicine [1]. Whether the de Winter ECG pattern can evolve into STEMI is debatable. Fiol Sala first proposed that an evolutionary ECG change between the de Winter ECG pattern and STEMI pattern was due to subtotal or total occlusion of the LAD. When a total occlusion of the anterior descending branch was not treated in a timely manner, it could evolve into STEMI. In addition, we believe that an evolutionary ST-T change in the ECG of patients with a de Winter ECG pattern could lead to a transformation from subtotal LAD occlusion (endocardial ischaemia) to total occlusion (transmural ischaemia), and the latter could cause a change from the de Winter ECG pattern to a STEMI. Therefore, we concur with Fiol Sala et al. that ST-segment elevation is possible without the coronary flow restoration [2, 4–6].

The lack of activation of sarcolemmal adenosine triphosphate (ATP) sensitive potassium (K_ATP_) channels was believed to cause the absence of ST-segment elevation in patients [1]. In the hyperacute phase of STEMI, tall, symmetrical T waves are caused by subendocardial ischaemia. In the acute phase of STEMI, ST-segment elevation is caused by transmural ischaemia [7]. However, the sensitivity to ischaemia is different between endocardium and epicardium, especially the cells at the junction between the intermediate myocardium and the endocardium. Hypoxia in the mid-myocardium may be the main cause of ECG manifestation of the de Winter pattern [8].

Some STEMI patients have a severe thrombus burden. Therefore, assessing their admission glucose is necessary because studies have shown that patients with admission hyperglycaemia would have increased mortality even if they underwent primary PCI [9]. One study reported that thrombus burden was associated with a pro-inflammatory/pro-coagulable state [10]. For these patients, thrombus aspiration (TA) was used to restore blood flow during the primary PCI; however, it was still unclear which type of hyperglyacemic STEMI patients were appropriate for TA [11].

## Conclusion

It is worth emphasizing that, regardless of the evolution of the de Winter ECG pattern, those ECGs represent acute coronary syndrome and should not be overlooked by emergency physicians and cardiologists. Similarly, the de Winter ECG pattern with stenocardia is equivalent to the STEMI pattern that should not be missed in the emergency setting. The evolutionary ECG changes depend on various factors, such as unstable thrombus in the proximal segment of the LAD, thrombus autolysis, and coronary artery recanalization. When the de Winter ECG pattern is observed, coronary recanalization could be necessary in order to open the culprit vessel and prevent the ECG from evolving into STEMI and irreversible myocardial damage.

## Data Availability

All available information is contained within the present manuscript.

## Abbreviations

LAD: Left anterior descending
ECG: Electrocardiogram
STEMI: ST-segment elevation myocardial infraction
ATP: Adenosine triphosphate
TA: Thrombus aspiration
